# Association of socioeconomic disadvantage with operative outcomes for infective endocarditis

**DOI:** 10.1371/journal.pone.0333221

**Published:** 2025-11-13

**Authors:** Barzin Badiee, Sara Sakowitz, Saad Mallick, Nguyen Le, Arjun Chaturvedi, Kevin Tabibian, Esteban Aguayo, Peyman Benharash

**Affiliations:** 1 Cardiovascular Outcomes Research Laboratories (CORELAB), David Geffen School of Medicine, University of California, Los Angeles, California, United States of America; 2 Division of Cardiac Surgery, Department of Surgery, David Geffen School of Medicine, University of California, Los Angeles, California, United States of America; University of Messina, ITALY

## Abstract

**Background:**

Social determinants of health (SDOH) are well-recognized contributors of disparities in cardiovascular disease. Yet, the association of socioeconomic disadvantage with outcomes following operative management of infectious endocarditis (IE) remains ill-defined.

**Methods:**

Nonelective adult (≥18 years) hospitalizations entailing cardiac valve operations for IE were tabulated from the 2016–2021 Nationwide Readmissions Database. Those facing adverse SDOH, including compromised economic, educational, healthcare, environmental, and social conditions, were categorized as Disadvantaged (others: Non-Disadvantaged). Multivariable regression models were developed to examine the association of socioeconomic disadvantage with key endpoints.

**Results:**

Of an estimated 36,527 hospitalizations, 31.9% were categorized as Disadvantaged with the proportion increasing from 27.0% in 2016 to 34.8% in 2021 (P < 0.001).

On average, Disadvantaged was younger (50 [34–64] vs 53 years [37–65], P < 0.001), more frequently insured by Medicaid (33.8 vs 26.4%, P < 0.001), and more commonly underwent isolated tricuspid operations (21.9 vs 13.7%, P < 0.001).

Following risk adjustment, Disadvantage remained linked with significantly greater likelihood of acute kidney injury (Adjusted Odds Ratio [AOR] 1.20, 95% Confidence Interval [CI] 1.12–1.29), stroke (AOR 1.25, 95%CI 1.11–1.41), and prolonged mechanical ventilation ≥24 hours (AOR 1.80, 95%CI 1.65–1.96). Further, Disadvantaged was associated with incremental increases in postoperative hospitalization duration (+4.97 days, 95%CI 4.39–5.54) and costs (+$32,900, 95%CI $29,300−36,500) as well as a greater risk of nonelective 90-day readmissions (AOR 1.12, 95%CI 1.03–1.23).

**Conclusions:**

Adverse SDOH are independently linked with greater morbidity and resource utilization following surgical management of IE. Efforts are needed to ensure comprehensive SDOH screening upon admission and develop targeted in-hospital interventions to address persistent disparities.

## Introduction

A growing body of work has linked adverse social determinants of health (SDOH) with significant barriers to complex cardiac care [1,2]. Considered to be the individual and structural factors that influence how individuals grow, live, and age [3], SDOH shape health literacy, engagement with preventative care, and access to medications as well as timely treatment [4]. These determinants encompass a broad range of needs, including financial hardship, housing instability, food insecurity, limited health literacy, psychosocial challenges, environmental risks, and barriers to accessing healthcare services [3]. Across a myriad surgical contexts, both personal and neighborhood socioeconomic disadvantage have been linked with greater likelihood of emergent presentation, higher morbidity, and increased resource utilization [5–7].

Adverse SDOH may be particularly impactful among patients with infective endocarditis (IE). A complex disease with mortality rates up to 50%, IE accounts for over $2.3 billion in hospitalization expenditures, each year [8,9]. However, beyond well-described links between intravenous drug use and IE incidence [10,11], reports have yet to thoroughly elucidate the association of social determinants with operative outcomes on a national scale. While a previous study by Strobel et al. [12]. reported an association between residence in socioeconomically distressed communities and increased mortality following valve operations, the impact of patient-level socioeconomic disadvantage on operative IE outcomes remains to be described.

As the number of operative cases for infective endocarditis continues to rise [8,13], a closer examination of surgical selection and management practices is warranted to guide quality improvement efforts. Therefore, we evaluated the association of SDOH with acute outcomes of operative intervention among a national cohort of patients presenting with acute IE. We hypothesized the presence of adverse SDOH to be independently associated with increased perioperative morbidity and resource utilization.

## Methods

All adult (≥18 years) nonelective hospitalizations requiring isolated aortic, mitral, pulmonic, tricuspid, or multi-valve procedures for IE, with or without coronary artery bypass grafting, were tabulated from the 2016–2021 Nationwide Readmissions Database (NRD) using previously-defined *International Classification of Diseases, Tenth Revision* (ICD) codes [6]. The NRD is the largest all-payer readmissions database in the US and provides accurate estimates for ~ 60% of all hospitalizations each year [14]. Given the fully de-identified nature of the NRD, this study was deemed exempt from full review by the Institutional Review Board at the University of California, Los Angeles.

To reduce cohort heterogeneity, patients undergoing transplant or aortic operations (6.3%), as well as those receiving mechanical circulatory support (1.4%) or percutaneous coronary intervention (0.3%), were excluded from further analysis. We additionally did not consider records missing data for age, sex, or in-hospital mortality (<0.1%).

**Fig 1:**
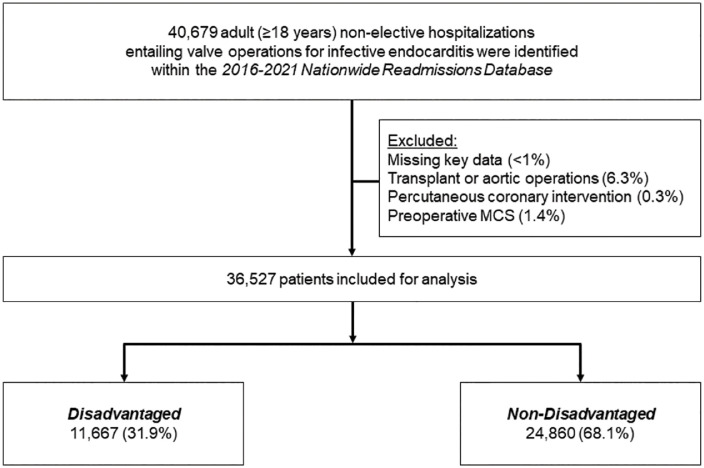
Study flowchart of survey-weighted estimates. Of an estimated 40,679 adult hospitalizations for cardiac surgery identified in the 2016-2021 Nationwide Readmissions Database, 36,527 patients were included for analysis. Of these, 11,667 (31.9%) were categorized as *Disadvantaged*. All estimates represent survey-weighted methodology. *MCS, Mechanical Circulatory Support.

The U.S. Department of Health and Human Services classifies SDOH based on economic, social, educational, healthcare, and environment-related parameters [3]. In line with this categorization, previously validated SDOH administrative codes were applied to identify relevant patient-level indicators [15]. These codes were categorized into the following subdomains: economic (housing instability, unemployment, food and financial insecurity), education (inadequate education, limited health literacy), social (psychosocial issues, racial disparities, legal challenges), healthcare (limited or inconsistent access to medical care), environment (exposure to hazards, unsafe living or work conditions) [15–17]. Prior work has documented that these codes accurately reflect patient social needs [18]. Consistent with previous methodology [15,16], patients with records containing at least one code indicating adverse SDOH were identified as socioeconomically disadvantaged and subsequently categorized as *Disadvantaged* (others: *Non-Disadvantaged*) (Fig 1).

Patient and hospital factors were captured using the Healthcare Cost and Utilization Project Data Dictionary [19]. The van Walraven modification of the Elixhauser Comorbidity Index was computed to ascertain patient burden of chronic illness [20]. Relevant comorbidities and complications were identified using previously-published ICD codes [21] We considered complications on the Society of Thoracic Surgeons short list, including acute kidney injury (AKI), infection, prolonged mechanical ventilation (≥24 hours), stroke, and reoperation during index admission. Hospitalization expenditures were calculated via application of institutional cost-to-charge ratios and adjusted for inflation using the 2021 Personal Healthcare Price Index [22].

The primary outcome of this study was in-hospital mortality. Secondary outcomes included perioperative complications, postoperative length of stay (pLOS), hospitalization costs, discharge disposition, and nonelective 30-day and 90-day readmissions.

Categorical variables are reported as proportions (%), while continuous variables are presented as medians with interquartile range (IQR) or means with standard deviation (SD). Bivariate comparisons were assessed using Pearson χ2, Mann–Whitney U and adjusted Wald tests, as appropriate. The significance of temporal trends was determined using the nonparametric Cochran-Armitage test [23].

To comprehensively adjust for intergroup differences, we utilized entropy balancing. This method searches for optimal sample weights to balance covariate distributions between groups [24]. Multivariable regression models were then constructed to evaluate the independent association between adverse SDOH and key study endpoints. Covariate selection was guided by the least absolute shrinkage and selection operator (LASSO), an automated method that improves model generalizability and reduces overfitting [25]. This approach was selected because it has been shown to yield robust and generalizable models relative to univariate analysis [26]. Outputs are reported as adjusted odds ratios (AOR) for logistic regressions and beta-coefficients (β) for linear regressions, respectively, both with 95% confidence intervals (95%CI). The threshold for statistical significance was established at α = 0.05. All analyses were conducted using Stata 18.0 (StataCorp, College Station, TX).

## Results

**Fig 2:**
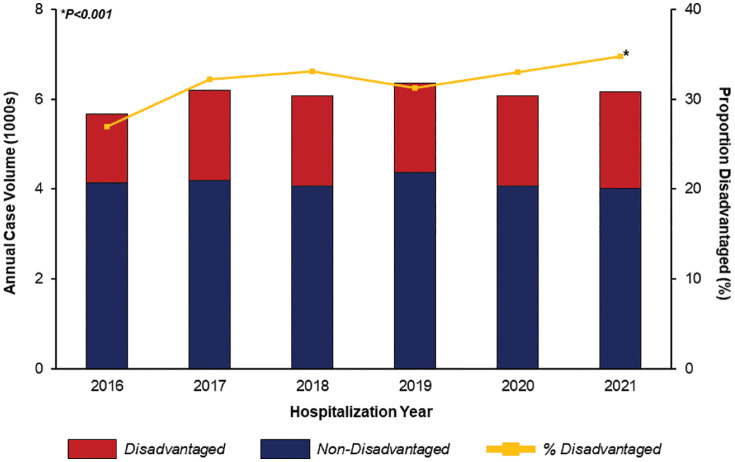
Trends in volume of valve operations for infective endocarditis. While the overall annual surgical volume was similar across years, the proportion of patients considered disadvantaged increased significantly over the study period. *Indicates statistical significance, P < 0.001.

Of an estimated 36,527 hospitalization records meeting inclusion criteria, 11,667 (31.9%) were categorized as *Disadvantaged*. The annual volume of valve operations for infective endocarditis was similar across the study period. However, the proportion of patients categorized as socioeconomically disadvantaged increased from 27.0% in 2016 to 34.8% in 2021 (P < 0.001, Fig 2).

On average, the *Disadvantaged* cohort was younger (50 [34–64] vs 53 years [37–65], P < 0.001), more commonly female (38.6 vs 33.6%, P < 0.001) and of a greater comorbidity burden (Elixhauser Index 4.5 ± 1.5 vs 4.1 ± 1.6, P < 0.001), compared to others. *Disadvantaged* also more frequently presented with a history of injection drug use (38.6 vs 28.7%, P < 0.001), liver disease (15.0 vs 11.1%, P < 0.001), and neurological disorders (20.1 vs 16.5%, P < 0.001). Moreover, *Disadvantaged* was more often insured by Medicaid (33.8 vs 26.4%, P < 0.001) or uninsured/self pay (13.6 vs 11.5%, P < 0.001). *Disadvantaged* patients more frequently underwent isolated tricuspid (21.9 vs 13.7%, P < 0.001) and multi-valve operations (20.4 vs 16.8%, P < 0.001), compared to *Non-Disadvantaged* (Table 1).

**Table 1:** Demographic, clinical, and hospital characteristics.

	*Non-Disadvantaged* (n = 24,860)	*Disadvantaged* (n = 11,667)	*P-value*
**Patient characteristics**			
Age (years, median [IQR])	53 [37-65]	50 [34-64]	<0.001
Female	33.6	38.6	<0.001
Elixhauser Index (mean ± SD)	4.14 ± 1.56	4.50 ± 1.48	<0.001
History of prior cardiac operation	12.9	11.2	0.003
*Cardiac procedure*			<0.001
Isolated aortic	33.1	24.3	
Isolated mitral	25.7	25.3	
Isolated pulmonic	0.8	0.4	
Isolated tricuspid	13.7	21.9	
Multi-valve	16.8	20.4	
CABG + valve	9.8	7.8	
*Median income percentile*			<0.001
76th - 100th	18.3	16.1	
51st - 75th	24.2	23.1	
26th - 50th	27.1	27.0	
0 - 25^th^	30.4	33.9	
*Insurance coverage*			<0.001
Private	29.0	21.6	
Medicare	33.1	31.1	
Medicaid	26.4	33.8	
Uninsured/self-pay	11.5	13.6	
*Comorbidities*			
Arrhythmia	43.5	37.2	<0.001
Coronary artery disease	17.5	10.6	<0.001
Chronic pulmonary disease	10.0	8.2	<0.001
Coagulopathy	24.5	26.5	0.01
Congestive heart failure	49.5	47.5	0.02
Injection drug use	28.7	38.6	<0.001
Liver disease	11.1	15.0	<0.001
Late-stage kidney disease	9.3	9.9	0.21
Neurological disorder	16.5	20.1	<0.001
Opioid use disorder	13.3	18.1	<0.001
Pulmonary circulatory disorder	22.9	29.6	<0.001
Tobacco use	45.9	45.0	0.31
**Hospital characteristics**			
*Hospital teaching status*			0.86
Non-metropolitan	1.3	1.4	
Metropolitan non-teaching	9.0	9.9	
Metropolitan teaching	88.9	88.7	

Reported as proportions unless otherwise noted. Statistical significance was set at α = 0.05.

*IQR, interquartile range; SD, standard deviation; CABG, coronary artery bypass grafting.*

On bivariate comparison, in-hospital mortality rate was similar between cohorts (7.3 vs 6.4%, P = 0.05). However, *Disadvantaged* demonstrated higher rates of AKI (43.7 vs 38.3%, P < 0.001), infection (3.3 vs 2.2%, P < 0.001), and stroke (10.2 vs 8.7%, P = 0.002). Additionally, *Disadvantaged* patients more often required prolonged mechanical ventilation (26.8 vs 17.4%, P < 0.001) and underwent reoperation during index hospitalization (3.1 vs 2.2%, P = 0.01). Evaluating resource utilization, patients experiencing adverse SDOH faced significantly longer pLOS (15 [8−28 days] vs 10 days [7−19 days], P < 0.001) and greater hospitalization costs ($115,900 [83,200−174,400] vs 94,000 [69,200−135,000], P < 0.001). *Disadvantaged* patients also commonly experienced nonelective 30-day (18.8 vs 17.5%, P = 0.04) and 90-day readmissions (33.6 vs 30.3%, P < 0.001) (Table 2).

**Table 2:** Unadjusted outcomes, stratified by the presence of adverse social determinants.

	*Non-Disadvantaged*	*Disadvantaged*	*P-value*
**Clinical Outcomes (%)**			
In-hospital mortality	6.4	7.3	0.05
Acute kidney injury	38.3	43.7	<0.001
Infection	2.2	3.3	<0.001
Prolonged mechanical ventilation	17.4	26.8	<0.001
Stroke	8.7	10.2	0.002
Reoperation	2.2	3.1	0.01
**Resource Utilization**			
pLOS (days, median, IQR)	10 [7–19]	15 [8-28]	<0.001
Costs ($1,000s, median, IQR)	94.0 [69.2-135.0]	115.9 [83.2-174.4]	<0.001
Nonhome discharge	41.5	50.7	<0.001
Nonelective 30-day readmission	17.5	18.8	0.04
Nonelective 90-day readmission	30.3	33.6	<0.001

Outcomes reported as proportions unless otherwise noted. Statistical significance was set at α = 0.05.

*IQR, interquartile range; pLOS, postoperative length of stay.

**Fig 3:**
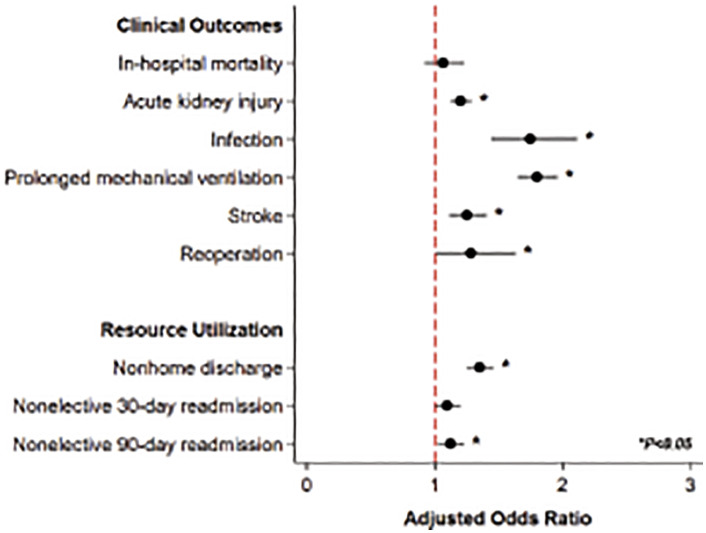
Association of disadvantaged status with perioperative outcomes. Disadvantaged status was associated with increased odds of acute kidney injury, infection, prolonged mechanical ventilation (≥24 hours), stroke, and reoperation during index hospitalization as well as greater resource utilization. *Indicates statistical significance, P < 0.05. Error bars represent 95% confidence intervals.

**Fig 4:**
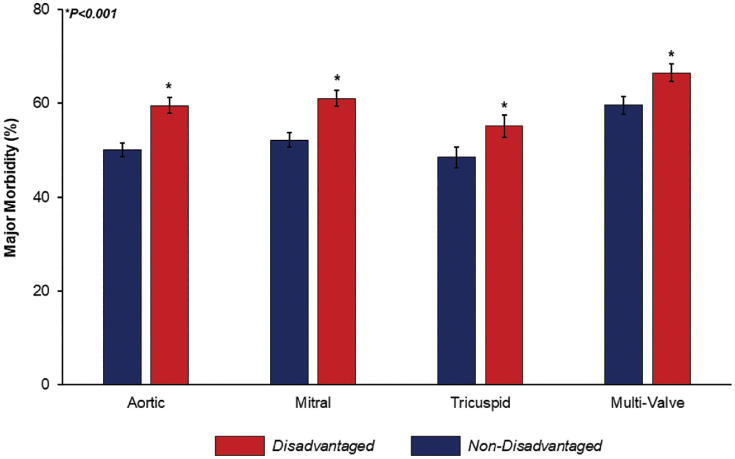
Adjusted risk of major morbidity, stratified by patient disadvantage and valve procedure. Across aortic, mitral, tricuspid, and multi-valve procedures, the presence of adverse social determinants of health was linked with elevated risk of major morbidity, comprising in-hospital mortality or any complication on the Society of Thoracic Surgeons short list. Patients faced greatest adjusted morbidity risk after multi-valve procedures (*Disadvantaged* 66.5% vs *Non-Disadvantaged* 60%). * indicates statistical significance, P < 0.001.

After entropy balancing and adjustment for baseline characteristics presented in Table 1, disadvantaged status remained was associated with similar odds of in-hospital mortality (AOR 1.06, 95%CI 0.92–1.23). Risk factors associated with in-hospital mortality are presented in Supplemental S1 Table. The presence of adverse SDOH was linked with increased odds of AKI (AOR 1.20, 95%CI 1.12–1.29), infection (AOR 1.75, 95%CI 1.44–2.11), stroke (AOR 1.25, 95%CI 1.11–1.41), prolonged mechanical ventilation (AOR 1.80, 95%CI 1.65–1.96), and reoperation during index hospitalization (AOR 1.28, 95%CI 1.01–1.63) (Fig 3). Stratifying by cardiac procedure, the *Disadvantaged* cohort faced increased likelihood of death or major morbidity across aortic, mitral, tricuspid, and multi-valve operations (Fig 4). Considering resource utilization, *Disadvantaged* patients demonstrated an incremental increase in adjusted pLOS (+4.97 days, 95%CI 4.39–5.54) and hospitalization costs (+$32,900, 95%CI $29,300−36,500). Moreover, *Disadvantaged* remained associated with a 35% increase in the relative risk of nonhome discharge (AOR 1.35, 95%CI 1.25–1.46). While risk of nonelective 30-day readmission did not achieve statistical significance, *Disadvantaged* was associated with a greater likelihood of nonelective 90-day readmissions (AOR 1.12, 95%CI 1.03–1.23) (Table 3). A subgroup analysis among patients without history of injection drug use demonstrated *Disadvantaged* to be associated with similar outcomes (Supplemental S2 Table). Further, a separate analysis using community-level income quartiles as the measure of socioeconomic disadvantage demonstrated attenuated associations, with fewer outcomes reaching statistical significance when comparing the lowest and highest quartiles (Supplemental S3 Table).

**Table 3:** Adjusted outcomes associated with adverse social determinants.

	Disadvantaged	95%CI	*P-value*
**Clinical outcomes**			
In-hospital mortality	1.06	0.92-1.23	0.39
Acute kidney injury	1.20	1.12-1.29	<0.001
Infection	1.75	1.44-2.11	<0.001
Prolonged mechanical ventilation	1.80	1.65-1.96	<0.001
Stroke	1.25	1.11-1.41	<0.001
Reoperation	1.28	1.01-1.63	0.04
**Resource Utilization**			
pLOS (β, days)	+4.97	4.39-5.54	<0.001
Costs (β, $1,000)	+32.9	29.3-36.5	<0.001
Nonhome discharge	1.35	1.25-1.46	<0.001
Nonelective 30-day readmission	1.10	1.00-1.20	0.05
Nonelective 90-day readmission	1.12	1.03-1.23	0.01

Outcomes reported as Adjusted Odds Ratio (AOR) or β Coefficient, with 95% confidence intervals (CI). Reference: *Non-Disadvantaged*.

*pLOS, postoperative length of stay.

## Discussion

Social determinants of health are well-established contributors to the incidence and severity of cardiovascular disease, as well as disparities in access to advanced care [1,2,4,6]. In the present study, we explored the impact of SDOH on operative outcomes for IE and made several key findings. First, we noted a significant increase in the proportion of socioeconomically disadvantaged patients who were surgically treated for infective endocarditis from 2016 to 2021. Additionally, the presence of adverse SDOH was independently associated with increased perioperative complications. Finally, socioeconomically disadvantaged patients faced greater duration of hospitalization and expenditures. With significant implications for both health policy and clinical management of these patients, several of these findings merit further discussion.

Our analysis highlighted a significant rise in the proportion of patients with documented adverse SDOH undergoing surgical management for IE. Specifically, we report a ~ 29% increase in the case volume of patients with unmet social needs from 2016 to 2021. Our work adds a new perspective to a growing body of literature documenting an upward trend in the incidence of IE among disadvantaged or unhoused patients [27,28]. Historically, socioeconomic disadvantage has been suggested to be an obstacle to valve surgery for IE [10,29]. Our study, therefore, may suggest that such patients are increasingly accepted as operative candidates, on a national level. However, our findings may also reveal that patients of adverse SDOH face considerable barriers to early IE diagnosis and appropriate antibiotic therapy, contributing to more severe symptom burden or systemic disease requiring surgical management [30–32]. Irrespective of cause, we found disadvantaged patients to be younger, more frequently have a history of injection drug use, and more commonly treated with tricuspid and multi-valve procedures. A growing body of literature has linked injection drug use (IDU) to IE [11,28,33,34], with the proportion of IDU-related IE cases having increased from 15% to 29% from 2010 and 2015 [33]. Strongly associated with socioeconomic vulnerability, IDU is more prevalent among younger patients and is a major risk factor for right-sided IE [11]. Our findings corroborate prior work which demonstrate an association between IDU and increased rates of tricuspid and multivalve surgical procedures in IE [11,28,34,35]. Together, these patterns describe an expanding population of surgical patients at high-risk for adverse outcomes and IE recurrence [12], for whom tailored management guidelines are needed. Implementing harm-reduction initiatives and integrating addiction medicine into multidisciplinary endocarditis teams may help address systemic and behavioral determinants of IE, supporting more sustainable management strategies [36].

Following comprehensive risk adjustment, socioeconomic disadvantage was associated with an increased risk of AKI, infection, stroke, prolonged mechanical ventilation, and reoperation during index admission. Building on a prior study linking residence in socioeconomically distressed communities with greater operative mortality [12], our findings highlight adverse patient-level SDOH as drivers of poor outcomes. Disadvantaged patients are documented to more frequently experience delayed presentation, which may stem from reduced access to primary care or timely cardiac evaluation [12]. This may result in more advanced infection by the time of surgical intervention, a factor which is associated with a greater risk of adverse events in surgically managed IE patients [37,38]. Further, the disadvantaged cohort more commonly required tricuspid valve or multi-valve operations, both of which have been associated with greater mortality [39]. Yet, the pervasive impact of socioeconomic disadvantage may also manifest through poor nutrition, physical inactivity, and chronic stress – factors that have been linked with impaired postoperative recovery [40–42]. Unfortunately, given the emergent nature of presentation in the case of IE, such factors are difficult to optimize in the hospital setting. Our findings, therefore, may instead reveal opportunities for perioperative care improvements. Screening checklists that incorporate social risk factors—such as housing instability, substance use, and limited support systems—have previously been explored in hospital settings to identify high-risk patients and may help tailor perioperative planning accordingly [43]. Incorporating SDOH into preoperative risk stratification models for IE may also help guide patient-centered decision-making and further address disparities in surgical access and outcomes [44]. Moreover, while surgical patients face baseline higher risk for recurrent IE [45], adverse SDOH may belie limited health literacy and barriers to access preventative antimicrobial therapy. While we could not examine IE recurrence in the present work, novel investigations are needed to consider medication adherence and long-term outcomes among this complex cohort.

We noted socioeconomically disadvantaged patients to incur significantly greater resource utilization, including a five-day increase in pLOS and an additional $32,900 in per-patient expenditures. Moreover, approximately 50% of patients with adverse SDOH required discharge to skilled nursing or acute rehabilitation facilities. Identification of unmet social needs for upcoming discharge disposition early in patients’ hospital course could permit better resource matching and eliminate any unnecessary delays [46]. Multi-disciplinary endocarditis teams should comprehensively evaluate patients’ needs at the onset of hospitalization, and ensure their access to adequate food and housing as well as medications and necessary rehabilitation services [36]. Standardized SDOH screening tools can further systematize this process, [47] guiding targeted referrals and potentially mitigating the higher risk of nonelective 90-day readmissions among disadvantaged patients. While SDOH screening requires considerable time and resources from care teams [48], comprehensive assessment of patient’s social needs has the potential to yield significant savings for the hospital and health system, through more efficient discharge planning and readmission prevention. In turn, institutions must ensure adequate staffing and support, so that care teams are not over-burdened, and SDOH screening efforts are sustainable in the long-term.

In a separate analysis, we used community-level income quartiles to define socioeconomic disadvantage. Interestingly, this approach yielded findings that differed from those based on the ICD-10 Z-codes used in the present study. When socioeconomic disadvantage was alternatively defined by community income quartiles, the associations with adverse outcomes were attenuated and less consistent, with several measures not reaching statistical significance. These divergent findings underscore the complexity of measuring disadvantage in administrative datasets. While Z-codes may capture individual-level social risk documented in the medical record, income quartiles represent a community-level proxy that may not fully reflect patient-specific vulnerability. Our results highlight the importance of how disadvantage is defined and suggest that future investigations should integrate multiple complementary measures to more comprehensively characterize the impact of adverse social determinants on operative outcomes in IE.

We acknowledge several important limitations. First, the NRD utilizes ICD coding, which can vary based on local billing practices. We were also unable to ascertain the timing of symptom onset relative to presentation for surgical management as well as the type of infective organism and adequacy of antibiotic therapy prior to surgery. The NRD similarly lacks detailed clinical variables such intensive care unit length of stay, timing to surgical intervention, and blood product use. Additionally, we could not ascertain the extent of hospitalization costs and length of stay associated with transfer to a nonhome facility. Furthermore, we utilized administrative codes to identify the presence of SDOH. While these codes have been previously validated to detect patients with unmet social needs, their utilization may still be limited in certain centers. This variability in the documentation of SDOH codes may contribute to underreporting and inconsistent identification of disadvantaged patients. Therefore, our findings may underestimate the association of SDOH with clinical and financial outcomes. Additionally, because there are no established ICD-10 codes specific to injection drug use, we relied on validated proxy codes described in prior work [49]. Importantly, we were unable to assess SDOH using indices such as the Social Vulnerability Index or the Distressed Communities Index, as these measures are not available within the NRD. Finally, we are unable to draw causative conclusions due to the retrospective nature of the study. Despite these limitations, we applied robust statistical methods to a large national cohort, to assess the association of socioeconomic disadvantage with operative outcomes for IE.

In conclusion, our study revealed a rise in the proportion of socioeconomically disadvantaged patients undergoing valve operations for IE. These patients faced increased perioperative complications and resource utilization. Our work suggests early screening for SDOH upon hospital admission may identify patients most at risk for adverse outcomes, and guide more informed perioperative management and discharge planning.

## Supporting information

S1 Table Risk-adjusted factors associated with in-hospital mortality. Outcomes reported as Adjusted Odds Ratio (AOR) with 95% confidence intervals (CI). *CABG, coronary artery bypass grafting.*
(DOCX)

S2 Table Adjusted outcomes associated with adverse social. determinants among patients without history of Injection drug use. Outcomes reported as Adjusted Odds Ratio (AOR) or β Coefficient, with 95% confidence intervals (CI). Reference: **Non-Disadvantaged.* pLOS, postoperative length of stay.*
(DOCX)

S3 Table Adjusted outcomes associated with lowest income quartile. Outcomes reported as Adjusted Odds Ratio (AOR) or β Coefficient, with 95% confidence intervals (CI). Reference: **Highest Income Quartile.* pLOS, postoperative length of stay.*
(DOCX)
