# Supplementary material for: Association of socioeconomic disadvantage with operative outcomes for infective endocarditis
Source: PLoS One. 2025 Nov 13;20(11):e0333221. doi: 10.1371/journal.pone.0333221 (PMC12614508; doi:10.1371/journal.pone.0333221)
Supplement: S1 Table — Outcomes reported as Adjusted Odds Ratio (AOR) with 95% confidence intervals (CI). CABG, coronary artery bypass grafting. (DOCX) [file pone.0333221.s001.docx]

**Supplemental Table 1:** Risk-adjusted factors associated with in-hospital mortality.

Outcomes reported as Adjusted Odds Ratio (AOR) with 95% confidence intervals (CI).

*CABG, coronary artery bypass grafting*

|  | **AOR** | **95%CI** | ***P-value*** |
| --- | --- | --- | --- |
| **Patient Characteristics** |  |  |  |
| Age (per year) | 1.03 | 1.02-1.03 | <0.001 |
| Female (reference: Male) | 1.14 | 0.99-1.31 | 0.07 |
| Elixhauser Index (per score) | 0.75 | 0.71-0.79 | <0.001 |
| Socioeconomic disadvantage | 1.09 | 0.94-1.26 | 0.25 |
| History of prior cardiac operation | 1.12 | 0.91-1.37 | 0.27 |
| *Cardiac Procedure* |  |  |  |
| Isolated aortic | Reference |  |  |
| Isolated mitral | 1.00 | 0.84-1.20 | 0.97 |
| Isolated pulmonic | 1.13 | 0.43-2.93 | 0.81 |
| Isolated tricuspid | 0.97 | 0.71-1.33 | 0.86 |
| Multi-valve | 1.81 | 1.51-2.17 | <0.001 |
| CABG + valve | 2.10 | 1.71-2.59 | <0.001 |
| *Median income percentile* |  |  |  |
| 76th - 100th | Reference |  |  |
| 51st - 75th | 1.08 | 0.88-1.32 | 0.46 |
| 26th - 50th | 1.39 | 1.15-1.69 | 0.001 |
| 0 - 25th | 1.18 | 0.97-1.43 | 0.11 |
| *Insurance coverage* |  |  |  |
| Private | Reference |  |  |
| Medicare | 1.24 | 1.03-1.49 | 0.02 |
| Medicaid | 1.26 | 1.02-1.57 | 0.03 |
| Uninsured/self-pay | 1.30 | 0.99-1.69 | 0.06 |
| *Comorbidities* |  |  |  |
| Arrhythmia | 0.98 | 0.86-1.13 | 0.78 |
| Coronary artery disease | 0.59 | 0.48-0.73 | <0.001 |
| Chronic pulmonary disease | 1.12 | 0.86-1.45 | 0.41 |
| Coagulopathy | 1.91 | 1.65-2.21 | <0.001 |
| Congestive heart failure | 1.59 | 1.38-1.84 | <0.001 |
| Injection drug use | 0.55 | 0.43-0.71 | <0.001 |
| Liver disease | 6.00 | 5.09-7.08 | <0.001 |
| Late-stage kidney disease | 2.69 | 2.21-3.26 | <0.001 |
| Neurological disorder | 2.06 | 1.76-2.41 | <0.001 |
| Opioid use disorder | 0.77 | 0.55-1.09 | 0.14 |
| Pulmonary circulatory disorder | 1.25 | 1.02-1.53 | 0.04 |
| Tobacco use | 0.69 | 0.59-0.80 | <0.001 |
|  |  |  |  |
| **Hospital characteristics** |  |  |  |
| *Hospital teaching status* |  |  |  |
| Non-metropolitan | Reference |  |  |
| Metropolitan non-teaching | 0.69 | 0.39-1.22 | 0.20 |
| Metropolitan teaching | 0.54 | 0.31-0.93 | 0.03 |
