# Supplementary material for: Association of socioeconomic disadvantage with operative outcomes for infective endocarditis
Source: PLoS One. 2025 Nov 13;20(11):e0333221. doi: 10.1371/journal.pone.0333221 (PMC12614508; doi:10.1371/journal.pone.0333221)
Supplement: S2 Table — determinants among patients without history of Injection drug use. Outcomes reported as Adjusted Odds Ratio (AOR) or β Coefficient, with 95% confidence intervals (CI). Reference: Non-Disadvantaged. pLOS, postoperative length of stay. (DOCX) [file pone.0333221.s002.docx]

**Supplemental Table 2:** Adjusted outcomes associated with adverse social determinants among patients without history of Injection drug use

Outcomes reported as Adjusted Odds Ratio (AOR) or β Coefficient, with 95% confidence intervals (CI). Reference: *Non-Disadvantaged*.

*pLOS, postoperative length of stay*

|  | **Disadvantaged** | **95%CI** | ***P-value*** |
| --- | --- | --- | --- |
| **Clinical outcomes** |  |  |  |
| In-hospital mortality | 1.12 | 0.95-1.31 | 0.18 |
| Acute kidney injury | 1.26 | 1.15-1.38 | <0.001 |
| Infection | 1.85 | 1.48-2.32 | <0.001 |
| Prolonged mechanical ventilation | 1.78 | 1.60-1.99 | <0.001 |
| Stroke | 1.23 | 1.07-1.41 | 0.004 |
| Reoperation | 1.47 | 1.12-1.92 | 0.005 |
|  |  |  |  |
| **Resource Utilization** |  |  |  |
| pLOS (β, days) | +5.88 | 5.13-6.63 | <0.001 |
| Costs (β, $1,000) | +40.4 | 35.6-45.2 | <0.001 |
| Nonhome discharge | 1.49 | 1.36-1.64 | <0.001 |
| Nonelective 30-day readmission | 1.06 | 0.95-1.18 | 0.29 |
| Nonelective 90-day readmission | 1.12 | 1.01-1.24 | 0.04 |
