# Supplementary material for: Association of socioeconomic disadvantage with operative outcomes for infective endocarditis
Source: PLoS One. 2025 Nov 13;20(11):e0333221. doi: 10.1371/journal.pone.0333221 (PMC12614508; doi:10.1371/journal.pone.0333221)
Supplement: S3 Table — Outcomes reported as Adjusted Odds Ratio (AOR) or β Coefficient, with 95% confidence intervals (CI). Reference: Highest Income Quartile. pLOS, postoperative length of stay. (DOCX) [file pone.0333221.s003.docx]

**Supplemental Table 3:** Adjusted outcomes associated with lowest income quartile.

Outcomes reported as Adjusted Odds Ratio (AOR) or β Coefficient, with 95% confidence intervals (CI). Reference: *Highest Income Quartile*.

*pLOS, postoperative length of stay*

|  | **Lowest Income Quartile** | **95%CI** | ***P-value*** |
| --- | --- | --- | --- |
| **Clinical outcomes** |  |  |  |
| In-hospital mortality | 1.05 | 0.83-1.32 | 0.69 |
| Acute kidney injury | 1.06 | 0.94-1.19 | 0.33 |
| Infection | 0.88 | 0.62-1.25 | 0.47 |
| Prolonged mechanical ventilation | 1.19 | 1.03-1.37 | 0.02 |
| Stroke | 0.90 | 0.75-1.09 | 0.28 |
| Reoperation | 1.18 | 0.84-1.67 | 0.33 |
|  |  |  |  |
| **Resource Utilization** |  |  |  |
| pLOS (β, days) | -0.28 | -1.19-(+0.63) | 0.54 |
| Costs (β, $1,000) | -22.5 | -28.2-(-16.7) | <0.001 |
| Nonhome discharge | 1.29 | 1.13-1.47 | <0.001 |
| Nonelective 30-day readmission | 0.97 | 0.83-1.12 | 0.65 |
| Nonelective 90-day readmission | 1.05 | 0.91-1.21 | 0.50 |
